# Supplementary material for: Risk factors for metabolic bone disease of prematurity: A meta-analysis
Source: PLoS One. 2022 Jun 13;17(6):e0269180. doi: 10.1371/journal.pone.0269180 (PMC9191712; doi:10.1371/journal.pone.0269180)
Supplement: S2 File — (DOCX) [file pone.0269180.s002.docx]

**Search strategy**

**Pubmed**

**(metabolic bone disease of prematurity[Title/Abstract]) AND (((((risk factor) OR (risk factors)) OR (risk)) OR (factor)) OR (relative risk))**
