# Supplementary material for: Risk factors for metabolic bone disease of prematurity: A meta-analysis
Source: PLoS One. 2022 Jun 13;17(6):e0269180. doi: 10.1371/journal.pone.0269180 (PMC9191712; doi:10.1371/journal.pone.0269180)
Supplement: S3 File — (DOC) [file pone.0269180.s003.doc]

**Data extracted from included studies**

1.birth weight

| number | literature | OR | OR_LL | OR_UL | Ln or | SE |
| --- | --- | --- | --- | --- | --- | --- |
| NO.2 | Meixi Wang 2021 [10] | 0.59 | 0.37 | 2.15 | -0.527632742 | 0.448908193 |
| NO.4 | Jiaxin Xu 2019 [12] | 0.62 | 0.389 | 0.99 | -0.478035801 | 0.238297347 |
| NO.5 | Meixi Wang 2019 [13] | 0.057 | 0.018 | 0.179 | -2.864704011 | 0.585972971 |
| NO.9 | Alejandro Avila-Alvarez 2020 [17] | 0.811 | 0.656 | 0.992 | -0.209487225 | 0.105500591 |

2.birth weight <1000g

| number | literature | OR | OR_LL | OR_UL | Ln or | SE |
| --- | --- | --- | --- | --- | --- | --- |
| NO.3 | Wei Wang 2020 [11] | 17.047 | 9.141 | 31.794 | 2.835974235 | 0.317986685 |
| NO.8 | Wenwen Chen 2021 [16] | 3.1 | 1.07 | 8.94 | 1.131402111 | 0.54155024 |
| NO.12 | Rios-Moreno 2016 [20] | 4.64 | 2.13 | 10.1 | 1.534714366 | 0.397044246 |

3.gestational age

| number | literature | OR | OR_LL | OR_UL | Ln or | SE |
| --- | --- | --- | --- | --- | --- | --- |
| NO.2 | Meixi Wang 2021 [10] | 0.63 | 0.45 | 0.72 | -0.46203546 | 0.119898885 |
| NO.5 | Meixi Wang 2019 [13] | 0.439 | 0.324 | 0.595 | -0.823255866 | 0.155055584 |
| NO.15 | Ebtihal Ali 2018 [23] |  |  |  | -0.41 | 0.19 |

4.gestational age <32 weeks

| number | literature | OR | OR_LL | OR_UL | Ln or | SE |
| --- | --- | --- | --- | --- | --- | --- |
| NO.1 | Xiaori He 2021 [9] | 2.358 | 1.358 | 4.095 | 0.857813802 | 0.281569818 |
| NO.3 | Wei Wang 2020 [11] | 6.521 | 5.707 | 7.450 | 1.875027738 | 0.067989934 |
| NO.10 | Wenhao Chen 2018 [18] | 1.498 | 1.127 | 1.991 | 0.404130885 | 0.145172906 |
| NO.14 | Ulf Ho¨gberg 2018 [22] | 2.3 | 1.04 | 5.07 | 0.832909123 | 0.404112272 |

5. Septicemia

| number | literature | OR | OR_LL | OR_UL | Ln or | SE |
| --- | --- | --- | --- | --- | --- | --- |
| NO.1 | Xiaori He 2021 [9] | 2.035 | 1.213 | 3.414 | 0.710495819 | 0.263976529 |
| NO.8 | Wenwen Chen 2021 [16] | 3.95 | 1.12 | 13.98 | 1.373715579 | 0.64395384 |
| NO.12 | Rios-Moreno 2016 [20] | 3.43 | 1.62 | 7.28 | 1.232560261 | 0.383343039 |

6. parenteral nutrition time

| number | literature | OR | OR_LL | OR_UL | Ln or | SE |
| --- | --- | --- | --- | --- | --- | --- |
| NO.3 | Wei Wang 2020 [11] | 3.473 | 2.252 | 5.356 | 1.245018774 | 0.221020081 |
| NO.4 | Jiaxin Xu 2019 [12] | 6.205 | 3.359 | 11.463 | 1.825355419 | 0.313132946 |
| NO.10 | Wenhao Chen 2018 [18] | 1.433 | 1.088 | 1.888 | 0.359770149 | 0.140606357 |
| NO.8 | Wenwen Chen 2021 [16] | 14.98 | 4.04 | 55.58 | 2.706715978 | 0.668770085 |

7.cholestasis

| number | literature | OR | OR_LL | OR_UL | Ln or | SE |
| --- | --- | --- | --- | --- | --- | --- |
| NO.3 | Wei Wang 2020 [11] | 2.071 | 1.656 | 2.589 | 0.728031582 | 0.113996593 |
| NO.8 | Wenwen Chen 2021 [16] | 4.44 | 1.59 | 12.40 | 1.490654376 | 0.523970014 |
| NO.11 | Supamit Ukarapong 2017 [19] | 9.6 | 2.1 | 45.3 | 2.261763098 | 0.783512675 |

8.intrauterine growth retardation

| number | literature | OR | OR_LL | OR_UL | Ln or | SE |
| --- | --- | --- | --- | --- | --- | --- |
| NO.3 | Wei Wang 2020 [11] | 5.778 | 2.602 | 41.034 | 1.754057602 | 0.703602197 |
| NO.5 | Meixi Wang 2019 [13] | 5.634 | 1.666 | 19.056 | 1.728819669 | 0.621672569 |
| NO.8 | Wenwen Chen 2021 [16] | 6 | 1.81 | 19.84 | 1.791759469 | 0.610809504 |
| NO.13 | Alicia Montaner Ramón 2017 [21] | 9.65 | 3.48 | 26.76 | 2.266957915 | 0.520376516 |
